# Myocilin mutations among POAG patients from two populations of Tamil Nadu, South India, a comparative analysis

**Published:** 2011-12-15

**Authors:** Rajiv Rose, Anandan Balakrishnan, Karthikeyan Muthusamy, Paramasivam Arumugam, Sambandham Shanmugam, Jayaraman Gopalswamy

**Affiliations:** Department of Genetics, Dr. ALMPGIBMS, University of Madras, Chennai, Tamil Nadu, India

## Abstract

**Purpose:**

Primary open angle glaucoma (POAG) is the most common type of glaucoma. Among the POAG genes identified so far, myocilin (*MYOC*) is the most frequently mutated gene in POAG patients worldwide. The *MYOC* Gln48His mutation is unique among Indian POAG patients. This mutation has not been observed in some populations within India and in other populations worldwide. The objectives of this work were to characterize and compare the mutation spectrum among POAG patients from two places of South India and identify the occurrence and prevalence of Gln48His mutation in our study populations.

**Methods:**

One hundred-one (101) POAG patients from Chennai, South India were recruited for the study. Earlier, 100 patients from the southernmost part of India, Kanyakumari district, were screened. *MYOC* was screened by polymerase chain reaction based single stand conformation polymorphism (PCR-SSCP) methodology. DNA sequencing of deviant samples was performed. Secondary structures of the proteins with amino acid sequence variations were predicted.

**Results:**

The mutation frequency of *MYOC* among POAG patients in Chennai was 2%. Three types of mutations were observed. The *MYOC* Gln48His mutation was observed among 2 POAG patients from Chennai. However, absence of this mutation among patients from Kanyakumari suggests possible involvement of demographic factors in disease causation via this mutation. Two heterozygous sequence variants, Thr353Ile and Asn480Lys, in the same exon (exon III) of *MYOC* were observed in one POAG patient who had a severe disease phenotype. This is the first such report of a compound heterozygote individual with two mutations in the same exon of *MYOC*.

**Conclusions:**

The presence of mutations at a rate similar to other studies suggests the causative role of *MYOC* among POAG patients from Chennai. Screening of more patients and families from all parts of India is required to identify the actual frequency of the Gln48His mutation and thus highlight its importance. The compound heterozygote with a severe disease phenotype reiterates the importance of *MYOC* in certain POAG patients.

## Introduction

Glaucoma is defined as progressive optic neuropathy that results in irreversible visual field loss [1,2]. Glaucoma is the second leading cause of blindness in the world [3]. Primary open angle glaucoma (POAG) is the most common type of glaucoma, affecting almost 2% of the world’s population [4]. India has a high prevalence of glaucoma with POAG being the most common type [5]. The prevalence of POAG in south Indian populations is about 1.6% [6,7] which is similar to that observed in Western populations.

Among the various risk factors, a positive family history of POAG is considered a major risk factor. Approximately, 16%–22% of first degree relatives of POAG patients develop the disease [8,9]. POAG is a complex heterogeneous disease from the number of loci, genes, and mutations involved. A recent review [10] lists 14 chromosomal loci for POAG as per HUGO (Human Genome Organization) [11-18]. Mutations in three genes namely, myocilin (*MYOC*, earlier called as the *TIGR*- Trabecular meshwork Inducible Glucocorticoid Response gene), optineurin (*OPTN*), and W (tryptophan) D (aspartic acid) repeat 36 (*WDR36*) have been identified among POAG patients [19-24]. Polymorphisms in cytochrome P450 1B1 (*CYP1B1*) [25,26] and heat shock protein 70-1 (*HSP 70-1*) [27] have been reported among POAG patients. Recently, the tumor protein 53 (*p53*) codon 72 polymorphism (variant rs1042522) was suggested to be an increased risk factor for POAG in Caucasians [28], while another study from Turkey did not find such an association [29]. Opticin (*OPTC*) as a possible candidate gene for POAG has been suggested [30].

Stone et al. [19] were the first to report mutations in *MYOC* among POAG patients. Since then, many sequence alterations have been reported in *MYOC* among POAG patients, worldwide [31-45]. Mutations in *MYOC* have been observed in the juvenile and adult onset forms of POAG. Mutations have also been reported among both familial and sporadic cases of POAG [19]. A few variants are recurrent in various populations world-wide while some mutations were observed to be ethnicity specific [46].

Mutation screening of a gene is normally performed for 2 reasons: a) to implicate the gene as a cause of a particular disease, and b) to identify as many mutations as possible for the purpose of understanding the genotype- phenotype relationships. It can be used to compare mutation profiles among different populations and for constructing practical genetic tests for clinical use [34]. Further, discovery of the range of mutation types and their locations may shed more light on the little known normal function of myocilin protein [47] and could contribute to the rapidly accruing knowledge on the pathogenic roles of *MYOC* in causation of POAG [2,47-49]. Identification of a specific mutation or mutations responsible for a patient’s disease will solidify diagnosis and may help predict clinical course of the disease [47].

An average mutation frequency of ~1.4 – 4.6% in *MYOC* among POAG subjects [4,32,35,38] has been reported from around the world. In studies on Indian patients, *MYOC* mutations have been observed at a frequency of ~7.1% [50], and 2% [51-53]. Most of the mutations (90%) have been observed to be present on exon III of *MYOC* [46,54]. The importance of exon I was highlighted by the observation of the Arg46Stop mutation which is second most common mutation, after Gln368Stop mutation. The Arg46Stop mutation has been reported only among Asians [46]. The importance of the exon I of *MYOC* among Indian POAG patients is evident due to the Gln48His variant. As of date the Gln48His has been reported only among POAG patients from India [50,52,55] and not from any other part of the world.

The Gln48His mutation has been reported among patients from Chennai (South India), Bangalore (South India), and Kolkata (Eastern India) [46,51,56]. However, this mutation was absent in patients from farther South of Chennai [49]. We also did not observe the Gln48His mutation in our mutation analysis of patients from Kanyakumari district (KK dist), South of Chennai [53]. We extended our mutation analysis to include POAG patients from Chennai to compare the mutation types and frequencies among the two places of South India.

## Methods

### Clinical samples

The institutional human ethical committee of Dr. ALMPGIBMS, University of Madras, Taramani Campus, Chennai, India, approved the study pattern. Informed consent was obtained from each individual before blood was drawn. One hundred patients from Kanyakumari district [53], and 101 patients from Chennai, diagnosed to have adult/juvenile-onset primary open angle glaucoma (JOAG) were recruited for the study. Among the 101 patients from Chennai, one of them (sample code; BSR-16) was a JOAG patient, native of Kolkata (eastern India), but was residing in Chennai. Among the 100 patients (native of Chennai), eighty seven were adult onset POAG and 13 were JOAG patients. Twenty two of the patients had at least one family member affected by POAG. In two cases the family history of the disease could not be confirmed. The patient, BSR-16, was included in the present study because of the early age of onset and severity of the disease. The patient also had a family history of the disease. Equal number of age, sex, and location matched controls were included in the study. Mean age of patients (n=201) at sample collection was 58.6±13.5 (mean±SD) years (Chennai- 59.8±14 years; KK district- 57.4±13.3 years), while that of controls (n=201; Chennai=101; KK district=100) was 58.9±14.4 years (Chennai- 60±14.8 years; KK district- 57.8±14.2 years). Among the individuals, 62.7% were males (Chennai- 70.3%; KK district- 55%) both among patients and controls. Proportion of male to female POAG patients were different when compared between Chennai (71 males, 30 females) and KK dist (55 males, 45 females); p=0.029 (Fisher’s exact test using GraphPad Prism version 5 for Windows; GraphPad Software, San Diego, CA). There were no other significant differences.

A complete ophthalmic examination which consisted of measurement of IOP, gonioscopic evaluation of the angle, examination and documentation of the optic disc, and the visual field (VF) testing was performed by certified ophthalmologists. Visual field testing was done using Humphreys automated field analyzer and/or Bjerrum’s tangent screen manual kinetic perimetry. Three or more contiguously missed points on automated perimetry tests (repeatedly) were taken as a VF defect [57]. Visual field examinations using Bjerrum’s (Tangent) screen, grading and reporting of the results were conducted by certified and experienced ophthalmologists. Localized arcuate area defects or greater, consistent with glaucomatous cupping and no other identifiable cause for the same were regarded as glaucomatous VF defects. The criteria for diagnosing glaucoma were based on earlier reports [53,58]. Classification of adult and juvenile types of POAG was based on the age of disease onset being ≥40 years or <40 years of age, respectively [2,58,59].

### Analysis of *MYOC*

Genomic DNA was isolated from the blood samples of patients and controls using the salting out method of Miller et al. [60] Eight primer sets (Table 1) were designed to amplify *MYOC* in fragments of <300 bp [53] for single strand conformation polymorphism (SSCP) analysis. PCR amplifications were performed in 20 µl reaction volumes containing ~100 ng of genomic DNA, 5 pmol of forward and reverse primers, 2 mM of each dNTP, 0.5 U of Taq polymerase with a standard buffer containing 1.5 mM MgCl_2_. Amplification was performed as per the conditions mentioned previously [53]. Briefly, these conditions include initial denaturation at 94 °C for 5 min, followed by 30 cycles of denaturation (94 °C, 30 s), annealing (primer pair dependent temperature - Table 1, 30 s), and extension (72 °C, 30 s) each, followed by a final extension of 2 min at 72 °C.

**Table 1:** Primer sequences used to amplify the *MYOC* gene in fragments of <300 base pairs along with primer name, annealing temperature (Ta) for each primer pair and the expected product size.

**Primer name**	**Sequence**	**Ta (degree Celsius)**	**Product Size (base pairs)**
RR3	Forward-5’-TGGATTAAGTGGTGCTTC-3'	58	227
RR4	Reverse-5’-TGGCTGATGAGGTCATAC-3’		
RR5	Forward-5’-GGATGTCCGCCAGGTTT-3’	60	260
RR6	Reverse-5’-CAATGTCCGTGTAGCCAC-3’		
RR7	Forward-5’-TGGCTACCACGGACAGTT-3’	62	243
RR8	Reverse-5’-GAGGTGTAGCTGCTGAC-3’		
RR9	Forward-5’-CCTTCATCATCTGTGGCA-3’	64	248
RR10	Reverse-5’-GTACAGCTTGGAGGCTT-3’		
RR1	Forward-5’-AGAGCTTTCCAGAGGAAG-3’	64	248
RR2	Reverse-5’-ATGACTGACATGGCCTGG-3’		
RR11	Forward-5’-GTCCCAATGAATCCAGCT-3’	62	268
RR12	Reverse-5’-TTGCTGTAGGCAGTCTCC-3’		
RR13	Forward-5’-GACCAGCTGGAAACCCA-3’	60	256
RR14	Reverse-5’-TGCTGAACTCAGAGTCC-3’		
RR15	Forward-5’-CATAGTCAATCCTTGGGC-3’	64	231
RR16	Reverse-5’-TAAAGACCACGTGGGCAC-3’		

The amplicon products were mixed with the loading dye (bromophenol blue + xylene cyanol + formamide + Na_2_EDTA) and double distilled water in the following ratio (2 μl + 2 μl + 16 μl; amplicon + dye + dd water), heat denatured for 5 min in boiling water and electrophoresed on a composite (acrylamide, bis acrylamide + agarose) gel [61] for 13 to 14 h. The percentage of the gels used and the electrophoresis voltage varied for amplicons of the different primers sets. Gels were silver stained as per the protocol of Bassam et al. [62] The amplicons with a mobility shift were reamplified, column purified and sequenced using dye termination chemistry and read using the 96 capillary 3730x1 DNA analyzer (Applied Biosystems, Carlsbad, CA). Sequences were analyzed using BLAST to identify the variation(s).

The DNA sequences with a variation were translated in silico to obtain the corresponding amino acid sequences. The amino acid sequences were subjected to the secondary structure prediction using the Garnier Osguthorpe Robson (GOR) prediction method [63,64]. Further, motifs and modifications sites were predicted using PROSITE and MotifScan.

## Results

A total of five different mutations and two polymorphisms were observed among patients from Kanyakumari district [53], and Chennai (Table 2). All the sequence changes have been submitted to dbSNP and accession numbers included in Table 2. There were no significant differences (p>0.05) between various cohorts with mutations versus those without mutations using Fisher’s exact test (contingency table analysis, GraphPad Prism version 5). Some of the cohort comparisons are listed below;

**Table 2:** Comparison of *MYOC* gene mutations and polymorphisms (with dbSNP accession numbers) observed among POAG patients from Chennai and Kanyakumari district of South India.

** **	** **	**Mutations**					**Polymorphisms**	
**Location**	**Group**	**Gln48His **ss295476454	**Ser331Thr **ss295476451	**Thr353Ile **ss295476455	**Pro370Leu **ss295476453	**Asn480Lys **ss295476456	**Thr325Thr **ss295476450	**Try347tyr **ss295476452
Chennai	Patients n=101	2	0	1	0	1	0	0
	Controls n=101	0	0	0	0	0	0	0
KK District	Patients n=100	0	1	0	1	0	1	4
	Controls n=100	0	0	0	0	0	0	0

1. Total number of patients with mutations (5 of 201) versus number of controls with mutations (0 of 201); p=0.06;
2. JOAG patients with mutations (2/27; including subject BSR-16) versus adult onset POAG patients with mutations (3/174), p=0.13;
3. Male patients with mutations (3/126) versus female patients with mutations (2/75), p=1;
4. Number of mutant Chennai patients (2/100; excluding subject BSR-16, not native of Chennai) versus number of mutant KK dist patients (2/100); p=1;
5. Chennai patients with mutations (2/100; excluding BSR-16, not native of Chennai) versus Chennai controls with mutations (0/101), p=0.24;
6. KK dist patients with mutations (2/100) versus KK dist controls with mutations (0/100), p=0.49.

All mutations observed among patients were in the heterozygous state and were absent in controls. Two different mutations, Pro370Leu and Ser331Thr, were observed in patients from Kanyakumari district, of which Ser331Thr was a novel variant. These two mutations, Pro370Leu and Ser331Thr, have been reported and discussed earlier [53] and hence are not discussed here. Three different mutations were observed among the 101 patients from Chennai.

### *MYOC* Gln48His

**Figure 1:**
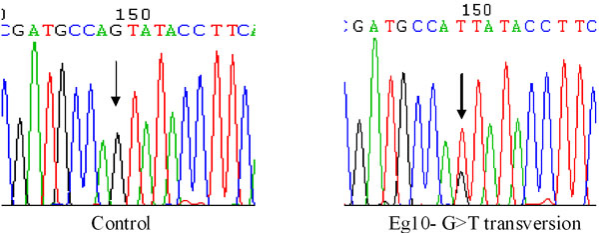
Heterozygous Gln48His mutation in *MYOC*. Chromatogram sequence derived from patient Eg-10 with the G>T transversion (indicated by arrow) compared to the normal control.

Two patients from Chennai (Sample Codes; Eg-17 and Eg-10) had the Gln48His variation (Figure 1). The Gln48His variation is a 144 G>T transversion which resulted in a non-conservative amino acid substitution, glutamine (Gln) to histidine (His). Presence of this mutation in patients and absence in controls indicates that this variant might play a disease causative role.

Age of onset of glaucoma was at 63 years for patient Eg-17. He had raised intraocular pressures and glaucomatous optic neuropathy (Table 3). Visual field tests revealed constricted fields in the right eye and tubular vision in the left eye. The patient was under medication - Iobet 0.5%. The patient Eg-10 had been diagnosed for POAG a year earlier (age of onset, 43). The patient had a raised IOP and increased cup/disc ratio in the right eye. The patient had an enlarged Blind Spot (BS) in the left eye and ≤35° vision in the right eye. The pressures were reduced to normal levels, 17.4 (OD)/7.2 (OS) mmHg after medication (Iobet 0.5%).

**Table 3:** Clinical details of South Indian POAG patients with *MYOC* mutations.

**Patient**	**Mutation**	**Age (at diagnosis)**	**GON- C/D ratio**		**BCVA**		**GVFD**		**IOP (mm Hg) at diagnosis**		**Medication/ Surgery**	**Family history**
** **	** **	** **	**RE**	**LE**	**RE**	**LE**	**RE**	**LE**	**RE**	**LE**	** **	** **
Eg-17	Gln48His	63	0.7	0.8	6/6	LPP	+	+	22	26	Medication	No
Eg-10	Gln48His	43	0.9	0.4	6/9	6/6	+	EBS	24.4	14.6	Medication	No
BSR-16*	Thr353Ile	14	0.9	1	6/9	NLP	+	Blind	21**	-	Surgery	Yes
BSR-16*	Asn480Lys	14	0.9	1	6/9	NLP	+	Blind	21**	-	Surgery	Yes
Ngl-12#	Ser331Thr	68	0.9	1	6/6	LPP	+	+	20.6	20.6	Medication	No
R-6#	ProLeu370	16	-	-	NLP	NLP	Blind	Blind	-	-	-	Yes

* - Undergone surgery when blood samples were collected at the age of 34. ** - IOP at sample collection (IOP data at diagnosis, not available). ^#^ - Published data [46]. Abbreviations: GVFD- Glaucomatous visual field defect; GON- Glaucomatous optic neuropathy; BCVA- Best corrected visual acuity, using Snellen chart, at time of sample collection; LPP- Light perception with projection; NLP- No light perception (blind); EBS- Enlarged blind spot.

The other patient with mutations (BSR-16, from Kolkata) turned out to be a compound heterozygote. Two mutations, Thr353Ile and Asn480Lys, both in the heterozygous state were observed in exon III of *MYOC*.

### *MYOC* Thr353Ile

**Figure 2:**
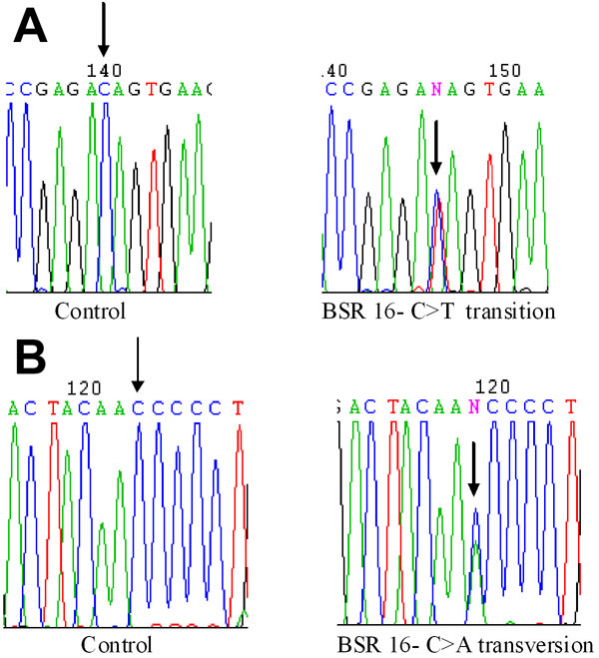
Chromatograms depicting *MYOC* gene sequence changes in a compound heterozygote POAG patient from India. **A**: Heterozygous The353Ile mutation in *MYOC*. Chromatogram sequence derived from patient BSR-16 with the C>T transition (indicated by arrow) compared to the normal control. **B**: Heterozygous Asn480Lys mutation in *MYOC*. Chromatogram sequence derived from patient BSR-16 with the C>A transversion (indicated by arrow) compared to the normal control.

This mutation is a heterozygous C>T transition in nucleotide 1058 (Figure 2A) which resulted in a threonine to isoleucine change. This results in change in polarity. This has been reported earlier among POAG patients and control subjects from Asia [4]. However in our study this was not observed in controls.

### *MYOC* Asn480Lys

This mutation, asparagine (polar amino acid) to lysine (positively charged amino acid), is a result of a heterozygous C>A transversion at nucleotide 1440 (Figure 2B). This variant has been reported from other populations [31]. Founder effect for this mutation has also been reported [31].

**Figure 3:**
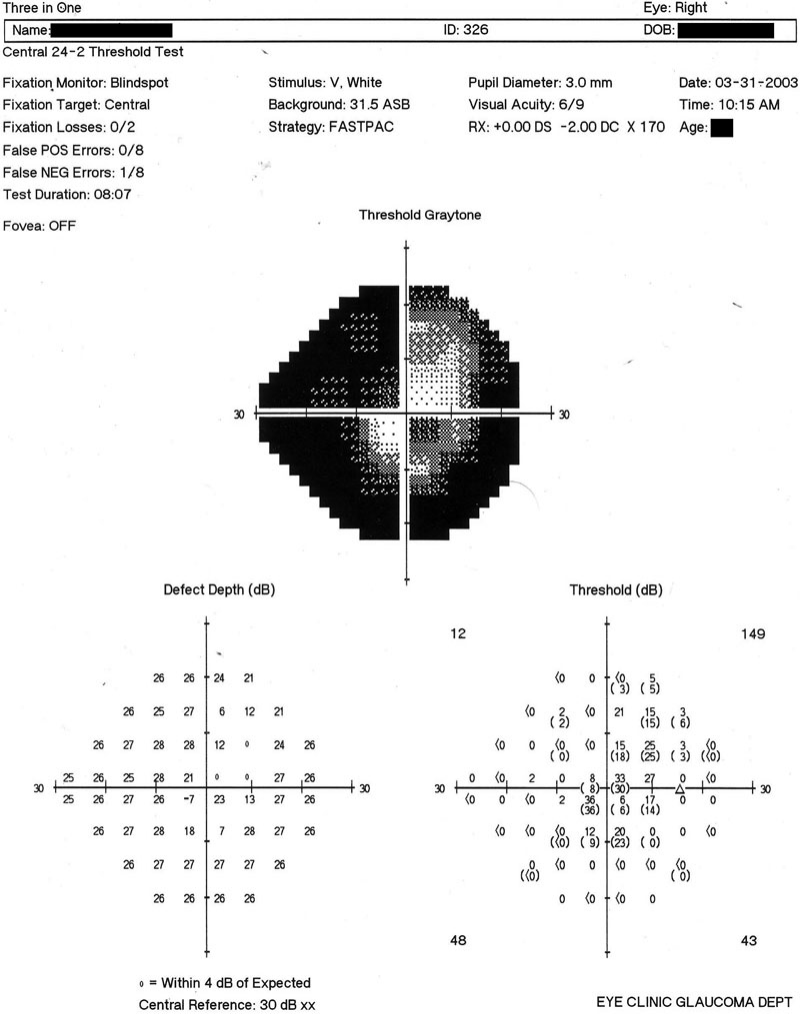
Humphreys visual field chart showing the visual field damage in the right eye of patient BSR-16, a compound heterozygote with two mutations in exon III of *MYOC*.

The patient BSR-16 had an early age of onset of the disease (diagnosed at age 14). She had rapid progression of the disease and before POAG could be diagnosed and treated (trabeculectomy) she had lost the vision of her left eye and had a considerable vision loss in the right eye. She had been diagnosed and treated elsewhere initially, before finally visiting one of the hospitals involved in the present study. The pressure in her right eye during the blood collection was 21 mmHg. The optic cup/disc ratio was 0.9 in the right eye with a totally excavated disc in the left eye (Table 3). The patient was blind in the left eye and had ~25° vision in the right eye (Figure 3). A positive family history of POAG was recorded based on the patient’s information.

Secondary structure prediction using GOR showed changes in the predicted structure among all the mutant protein sequences. Further, other tools to predict the modification sites namely PROSITE and MotifScan identified the presence of motifs/ modifications sites possibly being affected as a result of these secondary structural changes. The effects of the mutations based on the prediction using softwares have been shown in Table 4.

**Table 4:** Predicted effects of the observed *MYOC* mutations on secondary structure, putative motifs, and modification sites of the myocilin protein.

**Mutation**	**Predicted effect of mutation on secondary structure and on motifs/modification sites**
Gln48His	Loss of sheet, gain of turn near to the PKC site (44–46 amino acids)
Ser331thr	Change in predicted secondary structures of the mutant protein
Thr353Ile	Loss of PKC site at 353–355
Pro370Leu	Loss of turn near to CK site (377–380)
Asn480Lys	Change in charge, gain of α-helix at CK2 site (475–478)

Sequencing of 10 random samples using each primer set confirmed the results of SSCP analysis.

## Discussion

The mutation frequency in *MYOC* among POAG patients in this study was 2.48%. The mutation frequency observed among patients from Chennai was 2% (2 of 100; excluding the patient BSR-16 who originated from Kolkata, eastern part of India). A similar mutation frequency of 2% among POAG patients from kanyakumari district was reported earlier [53]. These observed mutation frequencies are similar to those reported from other populations in India [39,50,52,53] and rest of the world [4,38].

The presence of the *MYOC* Gln48His mutation among POAG patients from Chennai at a frequency of 2% conforms to earlier reports [39,52] and reiterates the importance and possible disease causing role of *MYOC*, especially exon I, among Indian POAG patients. The *MYOC* mutation frequency was much higher, 5.35% (3/56), in a study on POAG patients from Kolkata (eastern part of India) [50]. The frequency was lower at 0.8% in a study on patients from a different part of South India [56]. The Gln48His mutation has not been reported from any other population in the world so far.

Among patients from the KK district (the southern-most part of India) the Gln48His mutation was not observed [53]. In an earlier study by Kanagavalli et al. [51] the Gln48His mutation was not observed among patients from Madurai (farther south of Chennai and closer to the KK district). This suggests the possible involvement of demographic factors in causation of POAG (via the Gln48His mutation). Further, there are no reports of this variant being present in families. Inconsistency in the presence/absence of Gln48His and its prevalence among different populations in India makes it difficult to comprehend its role in disease causation.

It is essential to screen more patients from South India to confirm or rule out association of the Gln48His mutation with demographic factors. A proper mutation frequency is a prerequisite to initiate the screening of such region-specific mutations as a clinical diagnostic tool. Thus screening of Gln48His among POAG patients from other parts of India is also essential to establish it as a potential diagnostic tool for this part of the world. Since the Gln48His mutations have not been reported in familial cases from India, families with POAG must also be screened thoroughly to rule out or confirm the involvement of this mutation in familial cases.

Predictions using bioinformatics’ tools revealed changes in the secondary structure of the Gln48His mutant protein close to a putative phosphorylation (PKC) site at amino acids 44–46. Further, the cysteine at the 47th amino acid has been proposed to be involved in oligomerization of myocilin [65]. The predicted secondary structural changes due to the Gln48His mutation could hinder oligomerization via the cysteine residue (47th amino acid) thereby resulting in aberrant functioning of the protein. Functional studies are required to deduce the actual effect of the Gln48His mutation.

In this study two sequence variants in the same exon (exon III) of *MYOC* were observed in a POAG patient. The patient was a native of Kolkata (eastern part of India). The mutation frequency of *MYOC* is high among POAG patients of the eastern part of India [50]. Our study is the first report of a compound heterozygote with two mutations in the same exon (exon III) of *MYOC*. The occurrence of two variants (both resulting in amino acid changes) in different exons of the *MYOC* gene has been reported earlier [36] wherein the individual had a severe phenotype including an aggressive form of POAG and an early age of onset (14 years of age). The individual had a disease causing variation namely Lys423Glu (familial), and the Arg126Trp in exon I which had been observed in controls as well [36]. Two compound heterozygote POAG patients for the *CYP1B1* gene have been reported in one study [56].

Similar to the Arg126Trp in the earlier reported compound heterozygote [36], the Thr353Ile has been reported in controls [4] though not in our study and has been associated with an intermediate disease phenotype [4,66]. The other mutation Asn480Lys in our compound heterozygote has been reported in other studies as well and has also been associated with an intermediate disease phenotype [67,68]. Further, the Asn480Lys mutation has earlier been reported to be associated with families and to have a founder effect, (reviewed by Gong et al. [46]).

The compound heterozygote individual, BSR-16, had a positive family history and a severe disease phenotype. The patient, BSR-16, had an early age of disease onset (14 years of age) and aggressive form of the disease with rapid progression which was comparable to that reported earlier [36]. Efforts to screen the family for mutations proved futile as the members were not approachable (in terms of location and other factors) for blood samples. Presence of two variants in the same individual and the presence of a severe phenotype suggest a combinatorial role of the two variations in disease causation. This observation in the Kolkata patient, along with the earlier report of a high mutation frequency in *MYOC* of POAG patients from Kolkata [50], indicate the stronger association of *MYOC* with POAG in the eastern part of India compared with the other parts.

An early age of disease onset and rapid progression of the disease has also been observed in some patients with an apparently normal *MYOC* gene. These observations indicate the possible role of other genes in causation of POAG. However, in the case of patient BSR-16, the combinatorial effect of the 2 mutations cannot be ruled out. Both variants had either a predicted alteration of protein secondary structure that overlapped a putative phosphorylation (CK2) site or predicted alteration of a phosphorylation (PKC) site (Table 4). Functional analyses of the mutant proteins are essential to confirm the importance of these predicted alterations in putative modification sites or changes in secondary structure altering overlapping/ nearby motifs or modification sites.

Mutations in presently known genes account for only a low percentage of all POAG cases (less than 10%) [29]. About 5% of POAG is caused by mutations in myocilin or optineurin [47]. Hence, more extensive genetic studies are required to identify other genes involved and to understand the molecular mechanisms of the disease. Most cases of primary open angle glaucoma are complex and multi-factorial [47], hence the role of epigenetic processes such as DNA methylation and histone modifications along with small interfering RNA cannot be ruled out and have to be addressed to better understand the complete mechanism of disease onset and prognosis.

## References

[1] Gupta N, Weinreb RN (1997). New definitions of glaucoma.. Curr Opin Ophthalmol.

[2] Kwon YH, Fingert JH, Kuehn MH, Alward WLM (2009). Primary Open-Angle Glaucoma.. N Engl J Med.

[3] Kingman S (2004). Glaucoma is second leading cause of blindness globally.. Bull World Health Organ.

[4] Pang CP, Leung YF, Fan B, Baum L, Tong WC, Lee WS, Chua JKH, Fan DSP, Liu Y, Lam DSC (2002). TIGR/ MYOC gene sequence alterations in individuals with and without primary open-angle glaucoma.. Invest Ophthalmol Vis Sci.

[5] George R, Ve RS, Vijaya L (2010). Glaucoma in India: Estimated Burden of Disease.. J Glaucoma.

[6] Dandona L, Dandona R, Srinivas M, Mandal P, John RK, McCarty CA, Rao GN (2000). Open-angle glaucoma in an urban population in southern India: the Andhra Pradesh eye disease study.. Ophthalmology.

[7] Vijaya L, George R, Paul PG, Baskaran M, Arvind H, Raju P, Ramesh SV, Kumaramanickavel G, McCarty C (2005). Prevalence of open-angle glaucoma in a rural south Indian population.. Invest Ophthalmol Vis Sci.

[8] Wolfs RCW, Klaver CCW, Ramrattan RS, van Duijn CM, Hofman A, de Jong PT (1998). Genetic risk of primary open-angle glaucoma. Population-based familial aggregation study.. Arch Ophthalmol.

[9] Alward WLM (2000). The genetics of open-angle glaucoma: the story of GLC1A and myocilin.. Eye (Lond).

[10] Allingham RR, Liu Y, Rhee DJ (2009). The genetics of primary open-angle glaucoma: A review.. Exp Eye Res.

[11] Sheffield VC, Stone EM, Alward WL, Drack AV, Johnson AT, Streb LM, Nichols BE (1993). Genetic linkage of familial open angle glaucoma to chromosome 1q21-q31.. Nat Genet.

[12] Fan BJ, Wang DY, Lam DS, Pang CP (2006). Gene mapping for primary open angle glaucoma.. Clin Biochem.

[13] Sripriya S, Nirmaladevi J, George R, Hemamalini A, Baskaran M, Prema R, Ve Ramesh S, Karthiyayini T, Amali J, Job S, Vijaya L, Kumaramanickavel G (2006). OPTN gene: Profile of patients with glaucoma from India.. Mol Vis.

[14] Pang CP, Fan BJ, Canlas O, Wang DY, Dubois S, Tam PO, Lam DS, Raymond V, Ritch R (2006). A genome-wide scan maps a novel juvenile-onset primary open angle glaucoma locus to chromosome 5q.. Mol Vis.

[15] Wang DY, Fan BJ, Chua JK, Tam PO, Leung CK, Lam DS, Pang CPA (2006). Genome-wide Scan Maps a Novel Juvenile-Onset Primary Open-Angle Glaucoma Locus to 15q.. Invest Ophthalmol Vis Sci.

[16] Fan BJ, Ko WC, Wang DY, Canlas O, Ritch R, Lam DS, Pang CP (2007). Fine mapping of new glaucoma locus GLC1M and exclusion of neuregulin 2 as the causative gene.. Mol Vis.

[17] Sud A, Del Bono EA, Haines JL, Wiggs JL (2008). Fine mapping of the GLC1K juvenile primary open angle glaucoma locus and exclusion of candidate genes.. Mol Vis.

[18] Jiao X, Yang Z, Yang X, Chen Y, Tong Z, Zhao C, Zeng J, Chen H, Gibbs D, Sun X, Li B, Wakins WS, Meyer C, Wang X, Kasuga D, Bedell M, Pearson E, Weinreb RN, Leske MC, Hennis A, DeWan A, Nemesure B, Jorde LB, Hoh J, Hejtmancik JF, Zhang K (2009). Common variants on chromosome 2 and risk of primary open-angle glaucoma in the Afro-Caribbean population of Barbados.. Proc Natl Acad Sci USA.

[19] Stone EM, Fingert JH, Alward WLM, Nguyen TD, Polansky JR, Sunden SL, Nishimura D, Clark AF, Nystuen A, Nichols BE, Mackey DA, Ritch R, Kalenak JW, Craven ER, Sheffield VC (1997). Identification of a gene that causes primary open angle glaucoma.. Science.

[20] Rezaie T, Child A, Hitchings R, Brice G, Miller L, Coca-Prados M, Héon E, Krupin T, Ritch R, Kreutzer D, Crick RP, Sarfarazi M (2002). Adult-onset primary open-angle glaucoma caused by mutations in Optineurin.. Science.

[21] Monemi S, Spaeth G, DaSilva A, Popinchalk S, Ilitchev E, Liebmann J, Ritch R, Héon E, Crick RP, Child A, Sarfarazi M (2005). Identification of a novel adult-onset primary open-angle glaucoma (POAG) gene on 5q22.1.. Hum Mol Genet.

[22] Pasutto F, Mardin CY, Michels-Rautenstrauss K, Weber BH, Sticht H, Chavarria-Soley G, Rautenstrauss B, Kruse F, Reis A (2008). Profiling of WDR36 misense variants in German patients with glaucoma.. Invest Ophthalmol Vis Sci.

[23] Miyazawa A, Fuse N, Mengkegale M, Ryu M, Seimiya M, Wada Y, Nishida K (2007). Association between primary open-angle glaucoma and WDR36 DNA sequence variants in Japanese.. Mol Vis.

[24] Fan BJ, Wang DY, Cheng CY, Ko WC, Lam SC, Pang CP (2009). Different WDR36 mutation pattern in Chinese patients with primary open angle glaucoma.. Mol Vis.

[25] Stoilov I, Akarsu AN, Sarfarazi M (1997). Identification of three different truncating mutations in cytochrome P4501B1 (CYP1B1) as the principle cause of primary congenital glaucoma (buphthalmos) in families linked to the GLC3A locus on chromosome 2p21.. Hum Mol Genet.

[26] Bhattacharjee A, Banerjee D, Mookherjee S, Acharya M, Banerjee A, Ray A, Sen A (2008). Indian Genome Variation Consortium, Ray K. Leu432Val polymorphism in CYP1B1 as a susceptible factor towards predisposition to primary open angle glaucoma.. Mol Vis.

[27] Tosaka K, Mashima Y, Funayama T, Ohtake Y, Kimura I, Glaucoma Gene Research Group (2007). Association between open-angle glaucoma and gene polymorphism for heat-shock protein 70–1.. Jpn J Ophthalmol.

[28] Daugherty CL, Curtis H, Realini T, Charlton JF, Zareparsi S (2009). Primary open angle glaucoma in a Caucasian population is associated with the p53 codon 72 polymorphism.. Mol Vis.

[29] Saglar E, Yucel D, Bozkurt B, Ozgul RK, Irkec M, Ogus A (2009). Association of polymorphisms in APOE, p53, and p21 with primary open-angle glaucoma in Turkish patients.. Mol Vis.

[30] Acharya M, Mookherjee S, Bhattacharjee A, Thakur SK, Bandyopadhyay AK, Sen A, Chakrabarti S, Ray K (2007). Evaluation of the OPTC gene in primary open angle glaucoma: functional significance of a silent change.. BMC Mol Biol.

[31] Adam MF, Belmouden A, Binisti P, Brézin AP, Valtot F, Béchetoille A, Dascotte JC, Copin B, Gomez L, Chaventré A, Bach JF, Garchon HJ (1997). Recurrent mutations in a single exon encoding the evolutionarily conserved olfactomedin-homology domain of TIGR in familial open-angle glaucoma.. Hum Mol Genet.

[32] Wiggs JL, Allingham RR, Vollrath D, Jones KH, De La Paz M, Kern J, Patterson K, Babb VL, Del Bono EA, Broomer BW, Pericak-Vance MA, Haines JL (1998). Prevalence of mutations in TIGR/ Myocilin in patients with adult and juvenile primary open-angle glaucoma.. Am J Hum Genet.

[33] Rozsa FW, Shimizu S, Lichter PR, Johnson AT, Othman MI, Scott K, Downs CA, Nguyen TD, Polansky J, Richards JE (1998). GLC1A mutations point to regions of potential functional importance on the TIGR/ MYOC protein.. Mol Vis.

[34] Fingert JH, Héon E, Liebmann JM, Yamamoto T, Craig JE, Rait J, Kawase K, Hoh ST, Buys YM, Dickinson J, Hockey RR, Williams-Lyn D, Trope G, Kitazawa Y, Ritch R, Mackey DA, Alward WL, Sheffield VC, Stone EM (1999). Analysis of myocilin mutations in 1703 glaucoma patients from five different populations.. Hum Mol Genet.

[35] Yoon SJK, Kim HS, Moon JI, Lim JM, Joo CK (1999). Mutations of the TIGR/ MYOC gene in primary open-angle glaucoma in Korea.. Am J Hum Genet.

[36] Faucher M, Anctil J-L, Rodrigue M-A, Duchesne A, Bergeron D, Blondeau P, Côté G, Dubois S, Bergeron J, Arseneault R, Morissette J, Raymond V, Québec Glaucoma Network (2002). Founder TIGR/ myocilin mutations for glaucoma in the Québec population.. Hum Mol Genet.

[37] Ishikawa K, Funayama T, Ohtake T, Tanino T, Kurosaka D, Suzuki K, Ideta H, Fujimaki T, Tanihara H, Asaoka R, Naoi N, Yasuda N, Iwata T, Mashima Y (2004). Novel MYOC gene mutation, Phe369Leu, in Japanese patients with primary open-angle glaucoma detected by denaturing high-performance liquid chromatography.. J Glaucoma.

[38] Aldred MA, Baumber L, Hill A, Schwalbe EC, Goh K, Karwatowski W, Trembath RC (2004). Low prevalence of MYOC mutations in UK primary open-angle glaucoma patients limits the utility of genetic testing.. Hum Genet.

[39] Bhattacharjee A, Acharya M, Mukhopadhyay A, Mookherjee S, Banerjee D, Bandopadhyay AK, Thakur SK, Sen A, Ray K (2007). Myocilin variants in Indian patients with open-angle glaucoma.. Arch Ophthalmol.

[40] Xie X, Zhou X, Qu X, Wen J, Tian Y, Zheng F (2008). Two novel myocilin mutations in a Chinese family with primary open angle glaucoma.. Mol Vis.

[41] Zhuo YH, Wei YT, Bai YJ, Duan S, Lin MK, Saragovi HU, Ge J (2008). Pro370Leu MYOC gene mutation in a large Chinese family with juvenile-onset open angle glaucoma: correlation between genotype and phenotype.. Mol Vis.

[42] Mengkegale M, Fuse N, Miyazawa A, Takahashi K, Seimiya M, Yasui T, Tamai M, Nakazawa T, Nishida K (2008). Presence of myocilin sequence variants in Japanese patients with open-angle glaucoma.. Mol Vis.

[43] Qu X, Zhou X, Zhou K, Xie X, Tian Y (2010). New mutation in the MYOC gene and its association with primary open-angle glaucoma in a Chinese family.. Mol Biol Rep.

[44] Ennis S, Gibson J, Griffiths H, Bunyan D, Cree AJ, Robinson D, Self J, MacLeod A, Lotery A (2010). Prevalence of myocilin gene mutations in a novel UK cohort of POAG patients.. Eye (Lond).

[45] Whigham BT, Williams SEI, Liu Y, Rautenbach RM, Carmichael TR, Wheeler J, Ziskind A, Qin X, Schmidt S, Ramsay M, Hauser MA, Allingham RR (2011). Myocilin mutations in black South Africans with POAG.. Mol Vis.

[46] Gong G, Kosoko-lasaki O, Haynatzki GR, Wilson MR (2004). Genetic dissection of myocilin glaucoma.. Hum Mol Genet.

[47] Fingert JH (2011). Primary open-angle glaucoma genes.. Eye (Lond).

[48] Zhou Y, Grinchuk O, Tomarev SI (2008). Trangenic mice expressing the Tyr437His mutant of human myocilin protein develop glaucoma.. Invest Ophthalmol Vis Sci.

[49] Zode GS, Kuehn MH, Nishimura DY, Searby CC, Mohan K, Grozdanic SD, Bugge K, Anderson MG, Clark AF, Stone EM, Sheffield VC (2011). Reduction of ER stress via a chemical chaperone prevents disease phenotypes in a mouse model of primary open angle glaucoma.. J Clin Invest.

[50] Mukhopadhyay A, Acharya M, Mukherjee S, Ray J, Choudhury S, Khan M, Ray K (2002). Mutations in MYOC gene of Indian primary open angle glaucoma patients.. Mol Vis.

[51] Kanagavalli J, Krishnadas SR, Pandaranayaka E, Krishnaswamy S, Sundaresan P (2003). Evaluation and understanding of myocilin mutations in Indian primary open angle glaucoma patients.. Mol Vis.

[52] Sripriya S, Uthra S, Sangeetha S, George RJ, Hemamalini A, Paul PG, Amali J, Vijaya L, Kumaramanickavel G (2004). Low frequency of myocilin mutations in Indian primary open-angle glaucoma patients.. Clin Genet.

[53] Rose R, Karthikeyan M, Anandan B, Jayaraman G (2007). Myocilin mutations among primary open angle glaucoma patients of Kanyakumari district, South India.. Mol Vis.

[54] Markandaya M, Ramesh TK, Selvaraju V, Dorairaj SK, Prakash R, Shetty J, Kumar A (2004). Genetic analysis of an Indian family with members affected with juvenile- onset primary open angle glaucoma.. Ophthalmic Genet.

[55] Chakrabarti S, Kaur K, Komatireddy S, Acharya M, Devi KR, Mukhopadhyay A, Mandal AK, Hasnain SE, Chandrasekhar G, Thomas R, Ray K (2005). Gln48His is the prevalent myocilin mutation in primary open angle and primary congenital glaucoma phenotypes in India.. Mol Vis.

[56] Kumar A, Basavaraj MG, Gupta SK, Qamar I, Ali AM, Bajaj V, Ramesh TK, Prakash DR, Shetty JS, Dorairaj SK (2007). Role of CYP1B1, MYOC, OPTN and OPTC genes in adult-onset primary open angle glaucoma: predominance of CYP1B1 mutations in Indian patients.. Mol Vis.

[57] Wolfs RCW, Borger PH, Ramrattan RS, Klaver CVW, Hulsman CAA, Hofman A, Vingerling JR, Hitchings RA, de Jong PTVM (2000). Changing views on open-angle glaucoma: definitions and prevalences- the Rotterdam study.. Invest Ophthalmol Vis Sci.

[58] Morissette J, Côté G, Anctil JL, Plante M, Amyot M, Héon E, Trope GE, Weissenbach J, Raymond V (1995). A common gene for juvenile and adult-onset primary open-angle glaucomas confined to chromosome 1q.. Am J Hum Genet.

[59] Wiggs JL Lynch S Ynagi G Maselli M Auguste J Del Bono EA Olson LM Haines JL A genome wide scan identifies novel early-onset primary open-angle glaucoma loci on 9q22 and 20p12. Am J Hum Genet 2004 74 1314 20Epub 2004 Apr 23 15108121 10.1086/421533 PMC1182098

[60] Miller SA, Dykes DD, Polesky HF (1988). A simple salting out procedure for extracting DNA from human nucleated cells.. Nucleic Acids Res.

[61] Peng H, Du M, Ji J, Isaacson PG, Pan L (1995). High-resolution SSCP analysis using polyacrylamide agarose composite gel and a background free silver staining method.. Biotechniques.

[62] Bassam BJ, Caetano-Anolles G, Gresshoff PM (1991). Fast and sensitive silver staining of DNA in polyacrylamide gels.. Anal Biochem.

[63] Garnier J, Osguthorpe DJ, Robson B (1978). Analysis of the accuracy and implications of simple methods for predicting the secondary structure of globular proteins.. J Mol Biol.

[64] Garnier J, Gibrat JF, Robson B (1996). GOR method for predicting protein secondary structure from amino acid sequence.. Methods Enzymol.

[65] Fautsch MP, Johnson DH (2001). Characterization of myocilin- myocilin interactions.. Invest Ophthalmol Vis Sci.

[66] Lam DSC, Leung YF, Chua JKH, Baum L, Fan DS, Choy KW, Pang CP (2000). Truncations in the TIGR gene in individuals with and without primary open-angle glaucoma.. Invest Ophthalmol Vis Sci.

[67] Brézin AP, Adam MF, Belmouden A, Lureau MA, Chaventré A, Copin B, Gomez L, De Dinechin SD, Berkani M, Valtot F, Rouland JF, Dascotte JC, Bach JF, Garchon HJ (1998). Founder effect in GLC1A linked familial Open Angle Glaucoma in Northern France.. Am J Med Genet.

[68] Hulsman CAA, de Jong PTVM, Lettink M, Van Duijn CM, Hofman A, Bergen AA (2002). Myocilin mutations in a population -based sample of cases with open-angle glaucoma: the Rotterdam study.. Graefes Arch Clin Exp Ophthalmol.

